# Liver Cell Transformation in Chronic HBV Infection

**DOI:** 10.3390/v1030630

**Published:** 2009-10-30

**Authors:** Shirine Benhenda, Delphine Cougot, Christine Neuveut, Marie Annick Buendia

**Affiliations:** Oncogenesis and Molecular Virology Unit, Institut Pasteur, Inserm U579, 28 rue du Dr. Roux, 75724 Paris cedex 15, France; E-Mails: shirine.benhenda@pasteur.fr (S.B.); delphine.cougot@pasteur.fr (D.C.); christine.neuveut@pasteur.fr (C.N.)

**Keywords:** hepatitis B virus, hepatocellular carcinoma, HBx, pathogenesis, transformation

## Abstract

Epidemiological studies have provided overwhelming evidence for a causal role of chronic HBV infection in the development of hepatocellular carcinoma (HCC), but the molecular mechanisms underlying virally-induced tumorigenesis remain largely debated. In the absence of a dominant oncogene encoded by the HBV genome, indirect roles have been proposed, including insertional activation of cellular oncogenes by HBV DNA integration, induction of genetic instability by viral integration or by the regulatory protein HBx, and long term effects of viral proteins in enhancing immune-mediated liver disease. In this chapter, we discuss different models of HBV-mediated liver cell transformation based on animal systems of hepadnavirus infection as well as functional studies in hepatocyte and hepatoma cell lines. These studies might help identifying the cellular effectors connecting HBV infection and liver cell transformation.

## 1. Introduction

A number of viruses including the hepatitis B virus (HBV) have been recognized as human oncogenic agents [1,2]. The criteria accepted as establishing causality are based on (i) strong epidemiological association; (ii) finding of integrated or episomal viral genomes and gene products in the tumor cells, and (iii) directly oncogenic properties revealed by cell transformation in culture and production of tumors in experimental animals. However, the stringency of these criteria differs among the various malignancies. The case of HBV and hepatocellular carcinoma (HCC) provides one of the most convincing examples of causality based essentially on epidemiologic evidence, using the viral surface antigen HBsAg as specific viral marker of persistent infection [3,4]. Moreover, increased risk of developing HCC has been associated with HBsAg-negative HBV infection or occult infection [5,6]. Concerning the second criteria, integrated HBV DNA sequences and episomal HBV genomes have been found in a majority of HBV-related tumors [7,8]. The third criteria however, *in vivo* direct transforming capacities assessed in functional assays, is strikingly weak for HBV compared to other human tumor viruses. Thus, for HBV and HCC, as well as for hepatitis C virus (HCV) and HCC, etiological relation might be viewed as indirect, being caused by persistent viral replication and chronic tissue injury, rather than by inherent oncogenic features of the virus [2,9]. The role of the chronic necro-inflammatory disease induced by immune responses to HBV is largely recognized as a major cause of liver cancer. However, other studies have pointed to different procarcinogenic events induced more directly by the virus (reviewed in [10,11]). Notably, the hypothesis of a direct role of the virus is supported by the ability of HBV DNA to integrate into the genome of infected cells. Genomic changes and insertional activation of cancer-related genes have been shown to result from HBV DNA insertion into host chromosomes. Other data argue for a contribution of HBV gene products to the tumoral process. Notably, the regulatory protein HBx has been involved in disruption of cell cycle regulation, in activation of oncogenic pathways, as well as in induction of DNA damage and genetic instability, all features that underlie the liver tumorigenic process (reviewed in [12]).

In this article, we summarize some of the findings that support these notions and discuss the possible implications for our understanding of HBV-related tumorigenesis.

## 2. Animal Models of Hepadnavirus-Related Carcinogenesis

The human hepatitis B virus is the prototype of the hepadnavirus family, which includes closely related viruses infecting a limited number of primates, mammals and birds [13]. Mammalian hepadnavirus models have been extensively used for studying the molecular mechanisms leading to liver cancer [14], as well as for experimenting potential therapeutical approaches in the management of HBV infection [15]. In particular, woodchucks chronically infected with the woodchuck hepatitis virus (WHV) have provided a unique model in which viral DNA integration into the host genome plays a pivotal role in the tumoral process.

WHV-infected woodchucks develop chronic hepatitis and HCCs that are in many points similar to those associated with HBV infection in humans, although the liver of this rodent species is not susceptible to cirrhosis. In experimental inoculations shortly after birth, virtually all woodchucks that become chronic WHV carriers develop HCCs with a median tumor-free survival of 24 months and a median life expectancy of 30–32 months [16]. Moreover, HCC occurs in 17% of woodchucks serologically recovered from acute infection [17], and these tumors carry integrated viral sequences. These data may be explained by lifelong persistence of “occult” infection after recovery from acute hepadnavirus infection [18]. Related findings have been reported in human HCCs from HBsAg-negative patients [19], and it has been reported that occult HBV infection is a risk factor for HCC development [6]. By contrast, liver tumors are not seen in non-infected woodchucks over their entire life span. During preneoplastic stages, WHV replicates at high level in the liver and viral DNA is frequently integrated into host chromosomes, leading to clonal expansion of hepatocytes carrying integration events [20,21]. Early preneoplastic lesions consist of altered hepatic foci that overexpress N-myc and IGF-II [22]. At tumoral stage, almost all tumors carry integrated viral sequences that may be detected by Southern blotting, reflecting selection by clonal outgrowth of a transformed cell targeted by the integration event [14].

Search for oncogenes at integration sites in woodchuck HCCs has led us to demonstrate that WHV acts mainly as an insertional mutagen of *myc* family genes. The patterns of WHV DNA insertion in c-*myc* in these tumors share common aspects with those of Moloney murine leukemia virus (MLV) in murine T-cell lymphomas [23].

**Figure 1.**
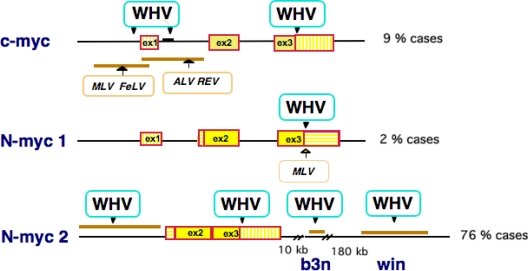
Insertional activation of *myc* genes in WHV-induced woodchuck HCCs. The c-myc, N-myc1 and N-myc2 loci are represented as a line with exons as boxes and WHV insertion sites are shown with arrows. Under each locus are shown the preferred integration sites of retroviruses including murine leukemia virus (MLV), feline leukemia virus (FeLV), avian leukemia virus (ALV) and avian reticuloendotheliosis virus (REV). The b3n and win loci are located at 10 and 180 kb downstream of N-myc2 on the woodchuck X chromosome. Percentages of WHV integration at each locus in a panel of 70 woodchuck tumors analyzed are shown on the right side.

The highest frequency of viral integrations was found in the woodchuck N-*myc* genes, particularly in the intronless retroposon N-*myc*2 and in two nearby loci on the X chromosome called *b3n* and *win* [24–28] (Figure 1). Interestingly, integrations targeting the N-*myc*2 gene have been found preferentially in large, advanced stage tumors, highlighting the strong oncogenic activity of N-*myc*2 [29]. Besides abnormal, elevated expression levels of the c-*myc* or N-*myc* oncogenes, evidence for a direct role of Myc activation in woodchuck tumorigenesis came from the finding that virtually all transgenic mice carrying WHV insertion sites in c-*myc* and N-*myc*2 developed HCC [30,31]. Myc is required for maintenance and expansion of cancer cells, where it hijacks different programs that drive normal cell proliferation. In murine models, Myc proved to be a key contributor to liver carcinogenesis, and recent studies have identified c-Myc as a central regulator at the early steps of malignant transformation in the human liver [32]. Thus, integration of WHV DNA confers a selective growth advantage on target hepatocytes, leading to the emergence of neoplastic nodules or providing an additional step in tumor progression.

The frequency of tumor incidence in humans and rodents is generally correlated with the fractional life span in a similar manner. The average life span of captive healthy woodchucks is about 10 years. Hepatocarcinogenesis appears therefore much more rapid and frequent in WHV-infected woodchucks than in human HBV carriers, suggesting that WHV might be a more oncogenic virus than HBV. As no insertional event into *myc* genes has been detected so far in human HCCs, the major difference in tumorigenicity between the two viruses might be attributed to the unique ability of WHV to provoke insertional activation of *myc* genes [33].

## 3. HBV DNA Integration into Human Chromosomes

In humans, HBV DNA integration into host chromosomes occurs since early stages in natural acute infections [34,35]. Multiple integrations have been detected in chronic hepatitis tissues [36,37], and integrated HBV sequences have been seen in most (about 80%) HBV-related HCCs [7,10]. In the absence of complete genomes in virtually all HBV inserts, these sequences cannot serve as template for viral replication. Integrated forms made of a single subgenomic fragment are believed to represent primary products of integration, and are frequent in HCC and hepatitis tissues from children [35]. Single HBV insertions are common in childhood HCCs but are rather uncommon later in life, suggesting that multiple integrations accumulate within single cells during long-standing HBV infections [38]. Studies of the organization of cloned HBV inserts in liver tissues and HCCs have shown that HBV sequences are often fragmented and rearranged. Moreover, the finding of integration and recombination sites dispersed over the viral genome suggests that HBV integration does not occur through a unique mechanism, as for integrase-mediated integration of retroviruses. However, highly preferred integration sites have been mapped in the HBV genome within the “cohesive ends” region, which lies between two 11-bp direct repeats (DR1 and DR2) highly conserved among hepadnaviruses [39]. More specifically, a narrow region encompassing DR1 has been shown to be particularly prone to recombination [40]. It encompasses a short terminal redundancy of the minus-strand DNA, which confers a triple-stranded structure to the circular viral genome. Accordingly, it has been proposed that relaxed circular DNA might be a preferential pre-integration substrate [41], and that Topo I might promote illegitimate recombination of hepadnavirus DNA *in vivo* [42].

In human HCCs, HBV DNA integration sites have been mapped to multiple regions on virtually all chromosomes, suggesting that viral integrations are distributed randomly throughout the host genome [35,43]. These events have multiple consequences either by inducing large-scale chromosome changes or by cis-acting effects on the expression or function of nearby cellular genes. Initial studies have suggested that HBV insertional mutagenesis is a rare event, and that simple repetitive elements such as Alu-type repeats, minisatellite-like, satellite III, or variable number of tandem repeat (VNTR) sequences are hotspots for HBV insertion in the human genome. However, evidence for a direct *cis*-acting promoter insertion mechanism was first provided in two independent HCCs. In one case, the HBV insertion targeted the retinoic acid receptor-ß (RARß) gene and resulted in the fusion of 29 amino-terminal residues of the HBV pre-S1 gene to the DNA-binding and hormone-binding domains of RARß [44,45]. Expression of the tumor-specific chimeric protein has been shown to display transforming potential [46]. In a second HCC, HBV DNA integration occurred in an intron of the human cyclin A gene, an essential gene for cell cycle progression, resulting in a strong expression of hybrid HBV/cyclin transcripts encoding a stabilized cyclin A [47,48]. Such modification of cyclin A expression has been linked to liver carcinogenesis [49]. In these two HCC cases, analysis of single HBV insertion sites has allowed identifying new genes that play critical roles in the control of cell growth and differentiation. Both studies have opened the way to novel approaches of the cellular pathways regulating cell division and differentiation.

More recently, the notion that HBV integrates randomly into human chromosomes has been challenged by large-scale analysis of HBV DNA integration sites using the Alu-PCR approach. These studies have revealed that targeting of cellular genes by HBV integration is a more frequent event than suspected previously [8,50]. As previously shown for retroviruses [51,52], HBV DNA integration appears to occur frequently in actively transcribed chromosomal regions, within genes or at their immediate vicinity. Recently, sequence analysis of 68 viral-host junctions from 60 HCCs provided evidence for cellular coding regions within several kbs in 90% of the cases [8]. Moreover, it was shown that HBV integration often targets gene families involved in cell survival, proliferation and immortalization, such as hTERT, PDGF receptor, MLL, calcium signaling-related genes and 60s ribosomal protein genes. Indeed, recurrent viral insertions nearby the hTERT or MLL gene have been reported by different groups [8,53–55]. Although the functional impact of integrated HBV sequences on nearby cellular genes has not been tested in all cases, these data support the view that viral insertion might be implicated in one of the multiple steps of liver cell transformation, and that different genes targeted by viral integration play important role in the tumoral process. Finally, although integrated viral sequences are defective for replication, they might also contribute “in trans” to tumorigenesis through the production of truncated and mutated HBx or preS2/S proteins. These proteins may act on HCC development by disrupting the control of cellular gene expression or by activating oncogenic signaling pathways.

Besides acting by cis–or trans-activation, HBV insertions have been associated with major genetic alterations within the cell genome, including large deletions, duplications and chromosomal translocations [56–60]. The association of HBV integration with large genomic changes might reflect the abrogation of control mechanisms that safeguard chromosomal integrity [61]. In this regard, it is interesting to note that HBV-related HCCs have been found to display higher rates of chromosomal alterations than HCCs related to other risk factors [62–64]. Moreover, molecular classification of HCC using genome-wide approaches has clearly indicated that HBV-related tumors harbor distinctive characteristics such as frequent mutations of the p53 tumor suppressor gene, activation of the mitotic cell cycle, deregulated expression of developmental and imprinted genes such as IGF2, and activation of the AKT pathway [64]. In addition, large-scale methodogies have led to conclude that HBV-related tumors are often poorly differentiated and present with unfavorable prognosis [65,66]. At preneoplastic stages, distinctive gene expression profiles have been detected in the non-tumoral livers of chronic hepatitis B carriers, such as activated expression of genes implicated in pro-apoptotic, inflammatory and DNA repair responses, suggesting specific oncogenic pathways triggered by chronic hepatitis B [67,68]. It seems therefore probable that HBV replication and integration might trigger various genomic changes in the infected hepatocytes, playing a role beyond the chronic necro-inflammatory disease induced by the host immune responses.

## 4. Oncogenic Capabilities of the HBx Regulatory Protein

**Figure 2.**
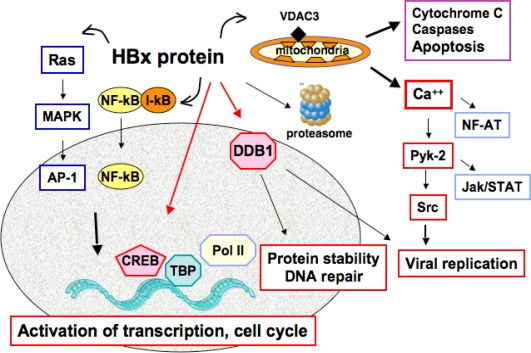
Pleiotropic activities of the regulatory protein HBx. Here we depict a large array of HBx functions that may be relevant for cellular transformation, such as stimulation of virus replication, cell cycle deregulation, activation of signaling pathways, induction/repression of apoptosis and interference with DNA repair. Most of these functions are achieved through the transcriptional transactivation activity of HBx or by interactions with cellular partners in both cell cytoplasm and nucleus (shown as a grey ellipse).

Among the viral gene products, the HBx protein encoded by the HBV X gene has been termed “viral oncoprotein” by many authors, and it has been involved in liver cell transformation because of its pleiotropic activities on cell cycle regulation, signaling pathways and DNA repair (reviewed in [69–71]) (Figure 2). However, different studies in cellular and animal models have provided divergent data on the transforming abilities of this viral protein. HBx has been reported to transform two cell lines immortalized by the simian virus 40 large T antigen (SV40TAg): Rev-2 and a mouse fetal hepatocyte cell line [72,73]. HBx has also been shown to cooperate with Ras in the transformation of primary human fibroblasts, either by activating the phosphatidylinositol-3 kinase and Akt pathway or by overcoming Ras-induced senescence [74,75]. A weak tumorigenicity has been attributed to HBx in TGF-α immortalized murine hepatocytes, but no cooperation could be evidenced with mutated p53 [76]. In contrast, other studies have shown that the apoptotic properties of HBx suppress transformation of primary rodent fibroblasts by different oncogenes [77]. One caveat with the observations described above is that most of them were made in cell culture under conditions of high levels of X gene expression. Indeed, in the chronically infected liver, HBx expression is kept at very low levels, rendering its detection uneasy with most available antibodies [78].

The oncogenic potential of HBx has also been addressed in transgenic mice, with again divergent results that probably reflect the use of different murine genetic backgrounds and various promoters yielding different expression levels of the viral protein. Development of HCC associated with high-level expression of HBx in the liver was essentially described for a transgenic mouse line generated in the outbred CD-1 background [79]. In other transgenic lines on different backgrounds, expression of HBx by itself did not lead to HCC development, although slight histopathologic alterations could be observed in the liver [80].

Transgenic expression of the viral protein cooperates with chemical carcinogens such as diethyl nitrosamine (DEN) in accelerating hepatocarcinogenesis [81]. By crossing HBx mice with WHV/c-myc oncomice, liver damage was increased and the average tumor latency was shortened by three months compared to WHV/c-myc littermates [82]. This could be explained by the reported ability of HBx to protect the c-Myc protein from degradation [83]. Moreover, HBx knock-in transgenic lines generated by homologous recombination into the mouse p21 locus develop HCC [84]. The CDK inhibitor p21^WAF1/Cip1^ is a master effector of tumor suppressor pathways that promote cell cycle arrest in response to various stimuli, and its genetic ablation might cooperate with HBx in cell transformation. Recently, it has been shown that constitutive liver expression of HBx and insulin receptor substrate-1 (IRS-1) contribute cooperatively to the development of dysplasia and eventually HCC [85]. The multisite docking protein IRS-1 alone had no direct oncogenic effects, but it stimulated hepatocyte proliferation through its central role in the transduction of growth factor signals, resulting in the activation of downstream mitogen-activated protein kinase (MAPK) cascade and the Wnt/β-catenin pathway in double transgenic mice. Together, these data suggest that HBx alone does not behave as a dominant oncogene but rather acts as a co-factor during hepatocarcinogenesis.

The mechanisms implicating HBx in the tumoral process have been investigated in HBx transgenic models. It has been shown that hepatic expression of HBx is associated with a significant increase in S-phase hepatocytes in young animals but not in adult mice [86], while our group observed increased levels of apoptosis that was independent of p53 in the transgenic livers [87]. These observations are consistent with a model in which HBx stimulates cell cycle progression of quiescent G0 cells to the G1/S phase, followed by prolongation of the G1-S transition or cell arrest at the G1/S boundary and eventually apoptosis [88–91]. A similar process might operate during liver regeneration after partial hepatectomy, leading to premature cell cycle entry or to impaired regeneration in different HBx transgenic lines [92–94]. Association with severe fat accumulation and impaired glycogen storage has been observed in the particular HBx transgenic line that eventually develop HCC [92], but not on other backgrounds. The pathological consequences of cell cycle deregulation by HBx have been extensively studied using established hepatocyte and hepatoma cell lines.

## 4. HBx, Replication Stress and Mitotic Defects

Recent studies have led to a consensus agreement on the fact that HBx interferes with the normal control of cell cycle progression. This function may be attributed to the ability of HBx to bind different cellular partners and to activate transcription as well as various signaling cascades [95–99]. The impact of HBx on the mitotic phase of the cell cycle seemingly implicates HBx in the induction of chromosomal instability (CIN), a hallmark of many tumors that correlates with the presence of extra centrosomes. It has been shown that CIN cells with extra centrosomes routinely undergo bipolar cell divisions, but display a significantly increased frequency of lagging chromosomes during anaphase [100]. Delayed movement (lagging) during anaphase results in chromosome missegregation, as some chromosomes or chromatids fail to be incorporated into one of the daughter nuclei following cell division. Accordingly, the observed prolongation of S phase in cells expressing the viral protein has been linked to aberrant centrosome duplication, multipolar spindle formation, chromosome segregation defects, and appearance of multinucleated cells.

Interestingly, in some studies these deleterious effects of HBx have been observed in the context of viral replication, and they were detectable in dividing, but not in quiescent hepatoma cells [101]. Different mechanisms have been implicated, in particular the interaction of HBx with UV-damaged DNA binding protein 1 (DDB1), a protein involved in DNA repair and cell cycle regulation [101]. The ability of HBx to bind DDB1, and the pivotal role of this interaction in viral infectivity and replication have been firmly established [102–104]. DDB1 is a subunit of the E3 ubiquitin ligase Cul4 complex that serves as an adaptor for selective ubiquitin-dependent degradation of target proteins [105,106]. It is known that anaphase onset and mitosis completion are highly complex and coordinated processes governed by dephosphorylation of Cdk substrates and ubiquitination of APC substrates. Deregulation of the mechanisms that control protein stability by the viral protein might therefore contribute to aberrant cellular growth and tumorigenesis.

Other studies have shown that HBx effects on centrosome dynamics and mitotic spindle formation are associated to the binding of HBx with different cellular partners implicated in centrosome formation. It has been reported that HBx interacts with HBXIP, a major regulator of centrosome duplication, required for bipolar spindle formation and cytokinesis. This interaction appears to be responsible for the formation of defective spindles and subsequent aberrant chromosome segregation [107]. In other report, HBx induces cytoplasmic sequestration of Crm1, a nuclear export receptor that binds to Ran GTPase and also localizes at the centrosomes. This interaction seemingly results in supernumerary centrosomes, increased frequency of defective mitoses and chromosome transmission errors [108]. Interestingly, recent data have linked DNA re-replication induced by HBx to partial polyploidy, known to be associated with cancer pathogenesis [109]. The mechanisms involve simultaneous increase of the replication initiation factors Cdc6 and Cdt1, and downregulation of geminin, the inhibitor of replication licensing. Modulation of Cdt1/geminin ratio leads to uncontrolled DNA re-replication, DNA damage, and partial polyploidy. The resulting polyploid cells are prone to oncogenic transformation that is dependent upon the expression of polo-like kinase 1 (Plk1), a crucial factor that positively influences mitotic entry [110]. HBx has also been shown to target BubR1, an effector of multiple mitotic kinases that specifies microtubule attachments and checkpoint functions [111]. It is worth noticing that mitotic phosphorylation of BubR1 by Plk1 is known to stimulate BubR1 kinase activity, promoting kinetochore microtubule attachments and recruitment of the Mad2 checkpoint protein to kinetochores. Moreover, it has been shown that SV40 large T antigen (LT) targets Bub1 and this has been correlated with alteration of the spindle checkpoint and oncogenic transformation.

Although the precise HBx activities that interfere with the mitotic cell cycle remain to be determined, these data provide insight into possible mechanisms leading to deregulation of the cellular mitotic machinery by a viral protein. They also provide a strong link between HBx expression and chromosomal instability in HBV-related carcinogenesis.

## Conclusions

Cancer arises from stepwise accumulation of genetic changes that confer unlimited, self-sufficient growth and resistance to normal homeostatic mechanisms. The long latency of HCC development after the primary HBV infection may be interpreted as a sign of an indirect action of the virus. It is generally admitted that host immune responses against infected hepatocytes triggers continuous necrosis and cell regeneration, leading to chronic inflammatory disease that in turn favors the accumulation of genetic alterations. In this view, long-term toxic effect of viral gene products, notably the HBx protein, as well as exogenous carcinogens such as aflatoxins and alcohol, potentiate the action of chronic liver inflammation. However, tumor onset might also depend on the occurrence of a decisive HBV integration event that would promote genetic instability or lead to *cis*- or *trans*-activation of cancer-related genes. The large variety of pathological properties attributed to the viral protein HBx indicates that multiple cooperative mechanisms may operate in the development of liver cancer. Future studies identifying more precisely the cellular effectors that mediate the tumorigenic effects of HBV are poised to advance the field of liver cancer biology and to develop adapted therapeutic strategies.
